# Esophageal Suspension Method for Hand-Sewn Esophagojejunostomy After Totally Laparoscopic Total Gastrectomy: A Simple, Safe, and Feasible Suturing Technique

**DOI:** 10.3389/fonc.2020.00575

**Published:** 2020-04-21

**Authors:** Chao Huang, Jiefeng Zhao, Zitao Liu, Jun Huang, Zhengming Zhu

**Affiliations:** Department of Gastrointestinal Surgery, The Second Affiliated Hospital of Nanchang University, Nanchang, China

**Keywords:** totally laparoscopic total gastrectomy, gastric cancer, esophageal suspension method, hand-sewn esophagojejunostomy, suture technique

## Abstract

**Background:** Totally laparoscopic total gastrectomy (TLTG) not only is difficult to operate but also has high technical requirements and a long learning curve. Therefore, it has not been widely carried out yet, and esophagojejunostomy is one of its difficulties. Relevant studies have shown that intracorporeal hand-sewn esophagojejunostomy is safe, feasible and low-cost, but it is complicated and time-consuming and requires a high-suture technique. This study introduces a simple, safe and feasible hand-sewn technique.

**Methods:** The clinical data of 32 patients with the esophageal suspension method for hand-sewn esophagojejunostomy (suspension group) after TLTG were collected from February 2018 to June 2019. During the same period, 32 patients with traditional hand-sewn esophagojejunostomy (traditional group) after TLTG were used as the control group.

**Results:** The operative time, anastomosis time, exhaust time and hospitalization time of the suspension group were shorter than those of the traditional group. The intraoperative blood loss in the suspension group was less than that in the traditional group. There were no postoperative complications associated with the suspension group.

**Conclusion:** For those who have some experience in laparoscopic suture technique, the esophageal suspension method for hand-sewn esophagojejunostomy after TLTG is a simple, safe, and feasible suture technique.

## Introduction

Since 1994, when Kitano et al. (1) first reported laparoscopic assisted distal gastrectomy (LADG) for early gastric cancer, laparoscopic technology has continuously matured and improved. At present, laparoscopic distal gastrectomy (LDG) is a way to treat gastric cancer, and its safety and feasibility have been indicated (2–4). However, totally laparoscopic total gastrectomy (TLTG) is a difficult operation, has high technical requirements and has a long learning curve. Therefore, it has not been widely carried out yet, and esophagojejunostomy is its main difficulty (5, 6). Roux-en-Y anastomosis is the main anastomosis of digestive tract reconstruction after total gastrectomy (7, 8). Currently, relevant literature (9–13) reports that esophagojejunostomy is mainly performed with circular and linear staplers. The former mainly includes the transorally inserted anvil (OrVil™), reverse puncture device (RPD), and purse-string suture method; the latter mainly includes functional end-to-end (FETE), overlap anastomosis, π-shaped esophagojejunostomy and semi-end-to-end anastomosis. Although the anastomosis technique has been continuously improved, there are still problems, such as difficulty in anvil implantation under the laparoscope, inaccurate esophageal cutting margins, and high price. In addition, related studies (14–17) have shown that intracorporeal hand-sewn esophagojejunostomy is safe, feasible and low-cost, but it is complicated and time-consuming and requires a high-suture technique. Therefore, it is particularly important to explore a simple, safe and feasible technique for hand-sewn esophagojejunostomy.

## Materials and Methods

### Patients

The clinical data of 32 patients with the esophageal suspension method for hand-sewn esophagojejunostomy (suspension group) after TLTG were collected from February 2018 to June 2019. During the same period, 32 patients with traditional hand-sewn esophagojejunostomy (traditional group) after TLTG were used as the control group. There were 32 patients in the suspension group, including 22 males and 10 females. The average age was 63.34 ± 9.86 years, and the average BMI was 21.80 ± 2.55 kg/m^2^. The tumor was located in the esophagogastric junction in 26 cases, including 10 cases of Siewert type II, 16 cases of Siewert type III, and in the middle of the stomach in six cases. The average follow-up time was 9.47 ± 2.83 months. There were 32 patients in the traditional group, including 24 males and eight females. The average age was 64.59 ± 10.90 years, and the average BMI was 22.42 ± 3.01 kg/m^2^. The tumor was located in the esophagogastric junction in 23 cases, including five cases of Siewert type II, 18 cases of Siewert type III, and in the middle of the stomach in nine cases. The average follow-up time was 9.34 ± 4.62 months (Table 1).

**Table 1:** Comparison of general characteristics and related indicators between the two groups of patients.

**Factors**	**Suspension group (*n* = 32)**	**Traditional group (*n* = 32)**	***P*-value**
Sex (*n*)			0.581
Male	22	24	
Female	10	8	
Age (y)	63.34 ± 9.86	64.59 ± 10.90	0.632
BMI(kg/m^2^)	21.80 ± 2.55	22.42 ± 3.01	0.378
Tumor location(n)			0.380
Esophagogastric junction	26	23	
Middle of stomach	6	9	
**Operation Indicators**			
Operation time (min)	185.81 ± 8.76	215.78 ± 8.08	<0.0001
Anastomosis time (min)	26 (23.25, 27)	44 (41.25, 45)	<0.0001
Intraoperative blood loss (ml)	98.28 ± 4.21	104.28 ± 5.51	<0.0001
**Postoperative Indicators**			
Exhaust time (*d*)	3 (3, 3)	3 (3, 4)	0.016
Hospitalization time (*d*)	11 (10, 12)	11.5 (11, 12)	0.006
**Postoperative Complications**			
Anastomotic leakage [*n* (%)]	0 (0)	1 (3.13)	1.000
Follow-up time (mon)	9.47 ± 2.83	9.34 ± 4.62	0.897

### Inclusion and Exclusion Criteria

The inclusion criteria of this study were as follows: 1. preoperative diagnosis was made by gastroscopy and pathology; 2. preoperative CT staging was T_1−2_N_0−1_M_0_; 3. patients had no history of abdominal surgery; 4. patients did not have neoadjuvant radiotherapy or chemotherapy before surgery; 5. TLTG was performed by the same treatment group; 6. patients signed informed consent; and 7. it was approved by the ethics committee of our hospital. We excluded patients with severe heart, lung, kidney, and brain dysfunction, as well as coagulopathy and intolerance.

### Operative Procedures

All patients underwent general anesthesia with tracheal intubation. A stomach tube and catheter were routinely inserted before operation. Patients were placed in the supine position with the two legs split. The operator stood on the left side of the patient, the first assistant stood on the right side of the patient, and the mirror holder stood between the patient's legs. The trocar was placed in a 5-hole method. After artificial pneumoperitoneum was established, a 10 mm trocar was placed under the umbilicus as the observation hole. The 5 mm trocars were placed in the left and right midline of the clavicle 2 cm above the umbilicus and 2 cm below the costal margin of the right anterior axillary line, respectively. The 12 mm trocar was placed 2 cm below the costal margin of the left anterior axillary line. The pneumoperitoneum pressure was maintained at 12–15 mmHg (1 mmHg = 0.133 kPa). Abdominal and pelvic conditions were routinely explored to rule out peritoneal implantation and distant metastasis. According to the requirements of radical gastrectomy, the perigastric vessels were isolated, and the corresponding lymph nodes were dissected with a harmonic scalpel. The duodenum and esophagus were cut with a linear cutting closure device, and the whole stomach specimen was taken through a semicircular incision around the umbilicus. The specimens were examined to ensure that the esophageal cutting edge was ~2 cm, and the upper and lower cutting edges were confirmed to be negative by frozen sections and reconstruction was started.

### Digestive Tract Reconstruction

**Figure 1:**
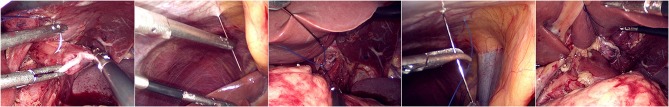
Esophageal right side suspension and fixation.

**Figure 2:**
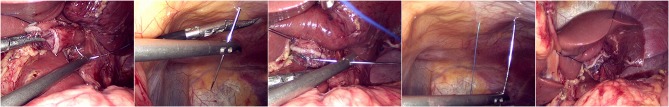
Esophageal left side suspension and fixation.

**Figure 3:**
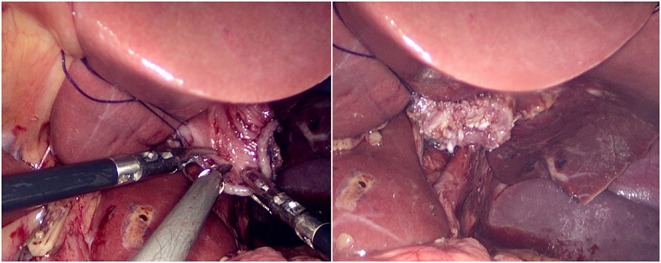
Harmonic scalpel dissected the proximal esophageal stump.

**Figure 4:**
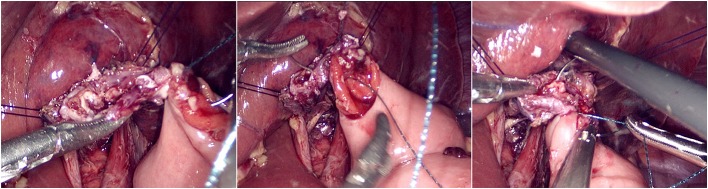
Continuous full-layer suture of the posterior wall of the esophagus and jejunum from left to right using 3-0 barbed line.

**Figure 5:**
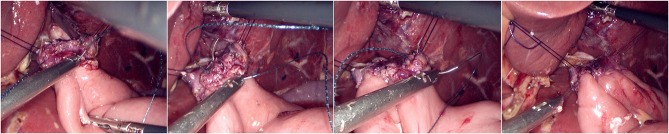
Continuous full-layer inverting suture of the anterior wall of the esophagus and jejunum from right to left using 3-0 barbed line.

**Figure 6:**
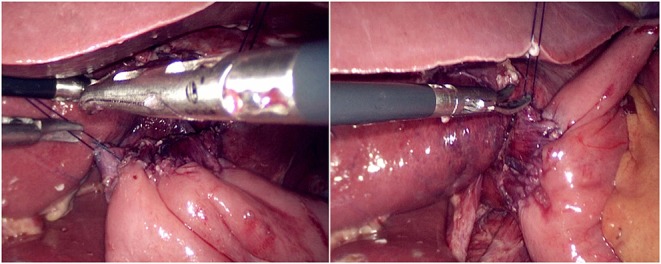
Cut left and right suspension suture.

**Figure 7:**
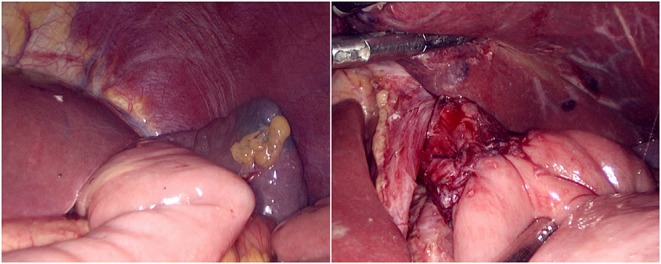
Placing the gastric tube into the distal jejunum and completing the esophagojejunostomy.

The jejunum was disconnected with a linear cutting closure device at a distance of 10–15 cm from the ligament of Treitz. The proximal jejunum and jejunum at a distance of 30–40 cm from the anastomosis of the esophageal jejunum was anastomosed end-to-side through the specimen incision. A small incision ~1.5–2 cm long was made at a distance of 3–5 cm from the distal jejunum to the stump and then put into the abdominal cavity. The pneumoperitoneum was established again. In the suspension group, the left and right sides of the proximal esophagus at a distance of 2 cm from the stump were suspended and fixed to the abdominal wall by the purse line or by the 3-0 Vichy line and purse line, respectively (Figures 1, 2). A harmonic scalpel was used to dissect the proximal esophageal stump (Figure 3). The distal jejunum was lifted posteriorly through the transverse colon, and the 3-0 barbed line was used to suture the posterior wall of the esophagus and jejunum with continuous full-layer suture from left to right (Figure 4) and to suture the anterior wall of the esophagus and jejunum with continuous full-layer inverting suture from right to left (Figure 5). After the anastomotic suture was completed, the suspension suture was cut off (Figure 6). The stomach tube was placed in the distal jejunum and esophagojejunostomy was completed (Figure 7). In the traditional group, hand-sewn esophagojejunostomy was performed directly without esophageal suspension.

### Observation Indicators

Operation indicators included operative time (from the insertion of the first trocar to the closure of the abdomen), anastomosis time (from the first suture to the end of anastomosis), and intraoperative blood loss. Postoperative recovery indicators included postoperative exhaust time and postoperative hospital stay. Postoperative complications included duodenal stump leakage, anastomotic leakage, anastomotic bleeding, anastomotic stenosis, obstructive complications, and perioperative mortality.

### Statistical Analysis

We used the single sample Kolmogorov-Smirnov test to test the normality of the data. If the quantitative data obeyed a normal distribution, it was described as the mean ± standard deviation; otherwise, it was described as the median and interquartile range. Quantitative data comparisons between the two groups were performed using the independent-samples *t*-test; otherwise, the Mann-Whitney *U*-test was used. Enumeration data comparisons between the two groups were performed using the chi-square test. A *P* < 0.05 was considered statistically significant. Data were analyzed using SPSS 22.0 for Windows (SPSS Inc., Chicago, IL, USA).

## Results

There were no statistically significant differences in sex (*P* = 0.581), age (*P* = 0.632), BMI (*P* = 0.378), tumor location (*P* = 0.380), or follow-up time (*P* = 0.897) between the two groups; there were statistically significant differences in operation time (*P* < 0.0001), anastomosis time (*P* < 0.0001), intraoperative blood loss (*P* < 0.0001), exhaust time (*P* = 0.016), and hospitalization time (*P* = 0.006) between the two groups (Table 1). The results showed that the operative time, anastomosis time, exhaust time and hospitalization time of the suspension group were shorter than those of the traditional group, and the intraoperative blood loss in the suspension group was less than that in the traditional group. There were no postoperative complications associated with the suspension group. There was no statistically significant difference in postoperative complications between the two groups (*P* = 1.000). One case in the traditional group developed an anastomotic leakage and recovered after conservative treatment. There were no other postoperative complications associated with either group (Table 1).

## Discussion

In recent years, LDG has been widely accepted and carried out in clinical practice as a safe and feasible method for the treatment of gastric cancer. Compared with laparotomy, LDG has advantages such as fast recovery of gastrointestinal function, short hospital stay, mild pain, small incision and fewer complications (18, 19). Meanwhile, the incidence of the adenocarcinoma of esophagogastric junction (AEG) is on the rise worldwide (20, 21). According to the Japanese gastric cancer treatment guidelines (22), except for early cancer, the retained stomach can be larger than 1/2, proximal gastric resection is used, and the rest should be considered for total gastrectomy. However, TLTG not only is a difficult operation but also has high technical requirements and a long learning curve. Therefore, it has not been widely carried out yet, and esophagojejunostomy is one of its difficulties. Laparoscopic total gastrectomy is currently performed mainly by laparoscopically assisted surgery, which is used to complete the reconstruction of the digestive tract through assisted small incision. However, for patients with a thick abdominal wall, large anterior and posterior diameter, narrow subcostal angle and left liver hypertrophy, it is difficult to complete anastomosis. If forced to complete the reconstruction, it is necessary to extend the incision and strengthen the traction, which not only loses the advantage of being minimally invasive but also increases the incidence of pain and anastomotic ischemia in patients (9, 10, 23). Therefore, to solve this problem, the development of TLTG is particularly important. Relevant studies (24, 25) reported that total laparoscopic distal gastrectomy (TLDG) has certain advantages compared with LADG, such as fast recovery of gastrointestinal function, short hospital stay, mild pain, and small incision. Previous studies (26–28) have also shown that total laparoscopic gastrectomy (TLG) and laparoscopic intracorporeal anastomosis were safe and feasible. In addition, TLG in intracorporeal anastomosis had the advantages of high safety, less adhesion, rapid recovery, and small scars (29, 30).

At present, the related literature (9–13) reports that esophagojejunostomy is mainly performed with circular and linear staplers. In fact, a variety of techniques for esophagojejunostomy have emerged in recent years, but none of them was considered as the standard technique (31). Although the anastomosis technique has been continuously improved, there are still problems, such as difficulty in anvil implantation under the laparoscope, inaccurate esophageal cutting margins, higher incidence of total anastomotic complications and high price (9, 32). Studies (33, 34) reported that the purse-string suture method was a safe and reliable technique. However, the long operation time, high operation difficulty and high price limit its application (35, 36). However, intracorporeal hand-sewn esophagojejunostomy under visualization can not only ensure the tension at the anastomosis but also make the anastomosis more reliable. More importantly, this method does not require a long esophageal stump and can significantly improve the R0 resection rate. Because the anastomosis was performed after the specimen was removed, a frozen section could be used to confirm the negative cutting edge for anastomosis. In addition, the hand-sewn esophagojejunostomy can reduce the use of the device, thereby reducing the cost of surgery. Related studies (14–17) have shown that intracorporeal hand-sewn esophagojejunostomy is safe, feasible and low-cost, but it is complicated and time-consuming and requires a high-suture technique. Facy et al. (37) believed that the incidence of anastomotic leakage, anastomotic bleeding, and anastomotic stenosis was low after laparoscopic hand-sewn esophagojejunostomy with a barbed line, so it was safe. In addition, the safety of barbed line sutures has also been demonstrated in large-scale trials (38, 39). This study used a 3-0 barbed line for esophagojejunostomy. Only one case developed an anastomotic leakage and recovered after conservative treatment. The esophageal suspension method for hand-sewn esophagojejunostomy can make suturing simple and easy. Relevant studies (40, 41) showed that increased operative time could significantly lengthen hospital stay for patients who underwent a primary laparoscopic Roux-en-Y gastric bypass. Meanwhile, Carter et al. (42) indicated that prolonged operating time can predict longer hospitalization after laparoscopic gastric bypass operation. In addition, short exhaust time makes patients eat early to accelerate the recovery of patients after surgery, thus shortening the hospital length of stay. This study showed that the operative time, anastomosis time, exhaust time and hospitalization time of the suspension group were shorter than those of the traditional group, and the intraoperative blood loss in the suspension group was less than that in the traditional group. There were no postoperative complications in the suspension group. Our experience was that, for Siewert type II AEJ, 3-0 Vichy line and purse line were used for esophageal suspension and fixation, while Siewert type III AEJ can be directly suspended and fixed by purse line. We believed that compared with the traditional hand-sewn method, the esophageal suspension method has the following advantages: 1. esophageal suspension and fixation on both sides can not only provide a stable suture field but also provide better exposure in the surgical field and reduce the secondary injury of tissues caused by repeated turnover of the esophagus and jejunum during operation; 2. the esophageal suspension method has lower requirements on the free length of the esophageal stump, which reduces the incidence of tissue bleeding; 3. the esophageal suspension method for hand-sewn esophagojejunostomy has relatively lower requirements for laparoscopic suture technique, which makes the learning curve relatively short and shortens the operation time. In addition, it should be noted that when the esophageal was suspended and fixed, the tearing of the esophageal wall caused by too shallow of an esophageal suture and excessive fixation force should be avoided. This study has some limitations. This study was a single-center, small-sample study, and therefore, a multicenter, large-scale prospective randomized controlled trial is necessary.

In conclusion, for those who have some experience in laparoscopic suture technique, the esophageal suspension method for hand-sewn esophagojejunostomy after TLTG is a simple, safe and feasible suture technique, which has an important reference value for the wide development of TLTG in the future.

## Data Availability Statement

All datasets generated for this study are included in the article/supplementary material.

## Ethics Statement

The studies involving human participants were reviewed and approved by Second Affiliated Hospital of Nanchang University. The patients/participants provided their written informed consent to participate in this study.

## Author Contributions

CH and ZZ designed the study. CH, JZ, and ZL collected clinical data. CH and JH analyzed the data. CH wrote the manuscript with contribution from all authors. All authors read and approved the final version of the paper.

## Conflict of Interest

The authors declare that the research was conducted in the absence of any commercial or financial relationships that could be construed as a potential conflict of interest.
